# Chemically Defined Organoid Culture System for Cholangiocyte Differentiation

**DOI:** 10.1002/adhm.202401511

**Published:** 2024-07-23

**Authors:** Zhenguo Wang, Shicheng Ye, Luc J.W. van der Laan, Kerstin Schneeberger, Rosalinde Masereeuw, Bart Spee

**Affiliations:** ^1^ Division of Pharmacology Utrecht Institute for Pharmaceutical Sciences Faculty of Science Utrecht University Universiteitsweg 99 Utrecht 3584 CG The Netherlands; ^2^ Department of Clinical Sciences Faculty of Veterinary Medicine Utrecht University Uppsalalaan 8 Utrecht 3584 CT The Netherlands; ^3^ Department of Surgery Erasmus MC Transplant Institute University Medical Center Rotterdam Doctor Molewaterplein 40 Rotterdam 3015 GD The Netherlands

**Keywords:** cholangiocyte, differentiation, intrahepatic cholangiocyte organoids, polarity, synthetic hydrogel

## Abstract

Cholangiocyte organoids provide a powerful platform for applications ranging from in vitro modeling to tissue engineering for regenerative medicine. However, their expansion and differentiation are typically conducted in animal‐derived hydrogels, which impede the full maturation of organoids into functional cholangiocytes. In addition, these hydrogels are poorly defined and complex, limiting the clinical applicability of organoids. In this study, a novel medium composition combined with synthetic polyisocyanopeptide (PIC) hydrogels to enhance the maturation of intrahepatic cholangiocyte organoids (ICOs) into functional cholangiocytes is utilized. ICOs cultured in the presence of sodium butyrate and valproic acid, a histone deacetylase inhibitor, and a Notch signaling activator, respectively, in PIC hydrogel exhibit a more mature phenotype, as evidenced by increased expression of key cholangiocyte markers, crucial for biliary function. Notably, mature cholangiocyte organoids in PIC hydrogel display apical‐out polarity, in contrast to the traditional basal‐out polarization of ICOs cultured in Matrigel. Moreover, these mature cholangiocyte organoids effectively model the biliary pro‐fibrotic response induced by transforming growth factor beta. Taken together, an animal‐free, chemically defined culture system that promotes the ICOs into mature cholangiocytes with apical‐out polarity, facilitating regenerative medicine applications and in vitro studies that require access to the apical membrane, is developed.

## Graphical abstract

Cholangiocyte organoids are crucial for disease modeling and regenerative medicine but face limitations with animal‐derived hydrogels and limited function. This study presents a synthetic polyisocyanopeptide hydrogel combined with an optimized medium to enhance cholangiocyte organoid maturation. The resulting organoids show improved functional markers, apical‐out polarity, and effectively model cholestasis and pro‐fibrotic responses, advancing their clinical application potential.

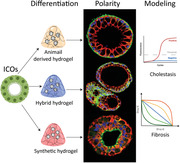

## 1 Introduction

Human intrahepatic cholangiocyte organoids (ICOs) are three‐dimensional (3D) tissue structures, generated from primary cholangiocytes which differentiate to progenitor/stem‐cell like cells with significant expansion potential in vitro. Notably, expanded ICOs have a bipotential differentiation capacity, and can be subsequently differentiated into either hepatocytes or cholangiocytes, making ICOs a promising tool for disease modeling,^[^ ^1^ ^]^ tissue engineering, as cellular building blocks for organs‐on‐a‐chip,^[^ ^2^ ^]^ and as a potential cell source for transplantation.^[^ ^3^ ^]^ However, differentiated ICOs remain immature with current culture protocols. Therefore, research efforts increasingly focus on novel ICOs differentiation methods in order to obtain more mature cholangiocytes or hepatocytes.^[^ ^2^, ^4^ ^]^

Currently, the most commonly used hydrogel for organoid generation and culture, including ICOs, relies on animal derived extracellular matrix (ECM) hydrogels. This native ECM hydrogel (e.g., Matrigel) is extracted from Engelbreth–Holm–Swarm mouse sarcoma cells, a tumor that produces many ECM proteins, and has been used for a myriad of organoid‐culture applications.^[^ ^5^ ^]^ However, these animal‐derived hydrogels exhibit significant lot‐to‐lot variations, in their biochemical properties, limited reproducibility. In addition, these hydrogels contain various growth factors and ECM components, such as laminins, which are essential for ICOs expansion^[^ ^6^ ^]^ but may hamper differentiation, making it difficult to control cell behavior and maturation. Moreover, when contemplating cell therapy, there is a potential risk of animal pathogen transmission and adverse immune responses, hampering clinical application of ICOs. It is therefore essential to find a replacement for animal‐derived hydrogels in order to promote the standardize of organoid cultures and facilitate regenerative medicine applications.

Synthetic hydrogels with defined physicochemical properties provide a promising tool to improve the reproducibility, standardization and utility of organoid cultures. From the early stages of organoid culture, several hydrogels, biological, semi‐synthetic or synthetic have been applied to organoid technology.^[^ ^7^ ^]^ For instance, as a biological hydrogel, Jagged1 immobilized on hyaluronic acid has been used for the generation of murine cholangiocyte organoids.^[^ ^8^ ^]^ Furthermore, several fully synthetic hydrogels have been developed for organoid culture. For instance, polyethylene glycol (PEG) and polyisocyanopeptides (PIC) modified hydrogels have demonstrated biocompatibility with liver organoid culture upon addition of cellular binding motifs.^[^ ^6^, ^9^ ^]^ Although these synthetic hydrogel‐based matrices were widely used for ICO differentiation toward hepatocyte‐like cells, their use in improving ICOs differentiation toward cholangiocytes has, as of yet, not been investigated.

In this study, we developed a novel differentiation media and utilized a thermo‐responsive PIC hydrogel to improve the differentiation of ICOs toward mature cholangiocyte. Previously, Kouwer et al. established a biomimetic material from PIC hydrogel with ECM‐mimicking properties.^[^ ^10^ ^]^ Subsequently, Zimoch et al. modified the PIC hydrogels to pre‐vascularize organotypic structures.^[^ ^11^ ^]^ In recent years, Ye et al. described a fully defined PIC hydrogel supplemented with laminin for human liver organoid expansion and differentiation into hepatocyte‐like cells.^[^ ^6^ ^]^ In addition, RNA sequencing of salivary gland organoids indicated a more differentiated phenotype in PIC compared to the standard Matrigel culture.^[^ ^12^ ^]^ Here, we propose a novel differentiation media in combination with the PIC hydrogel as a suitable alternative to native hydrogels due to its superior chemically defined and thermo‐reversible gelation,^[^ ^13^ ^]^ with the aim to improve the cholangiocyte differentiation of ICOs. To demonstrate the utility of our novel culture system, we applied it to study bile acid homeostasis and sensitivity to bile duct injury, as well as disease modeling of bile duct fibrosis. We envision that a fully defined, animal‐free culture system with more mature cholangiocyte organoids could be used in the future for liver transplantation and preclinical drug screening.

## 2 Results

### 2.1 Optimization of ICO Culture Media for Cholangiocyte Maturation

We have previously reported a novel method to differentiate ICOs into cholangiocyte‐like‐cells (CLCs) utilizing a mixed natural hydrogel (Matrigel mixed with collagen type I).^[^ ^2^ ^]^ To optimize the culture conditions and promote further maturation of ICOs, we investigated the addition of small molecules within the natural hydrogel before transitioning to the synthetic hydrogel. For this, two compounds were used to inhibit hepatocyte differentiation and promote a maturation of the cholangiocyte phenotype, namely, sodium butyrate (SB) and valproic acid (VPA), a histone deacetylase inhibitor and active Notch signaling activator, respectively.^[^ ^14^ ^]^ ICOs were exposed to these compounds for 7 d during differentiation with concentrations ranging from 1 to 4 × 10^−3^ m. Gene expression profiling after exposure to the compounds indicated that sodium butyrate and valproic acid have the ability to reduce hepatocyte markers expression, such as albumin (*ALB*), alpha fetoprotein (*AFP*), and hepatocyte nuclear factor 4 alpha (*HNF4A*), and upregulate cholangiocyte markers including Hes related family bHLH transcription factor with YRPW motif 1 (*HEY1*), secreted phosphoprotein 1 (*SPP1*), ATP binding cassette subfamily B member 1 (*ABCB1*), solute carrier family 10 member 2 (*SCL10A2*), and somatostatin receptor 2 (*SSTR2*), as shown in Figure S1 (Supporting Information). Both compounds enhanced cholangiocytes maturation, and as trends were visible, we used medium supplemented with 1 × 10^−3^ m sodium butyrate and valproic acid as an improved cholangiocyte differentiation medium (CDM) to drive the ICOs toward a more mature cholangiocyte phenotype.

**Figure 1:**
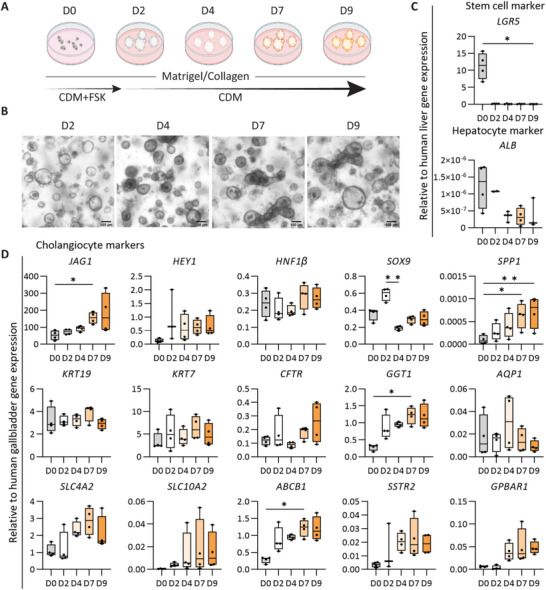
Improved differentiation of human intrahepatic cholangiocyte organoids (ICOs) to mature cholangiocyte phenotype. A) Schematic of the protocol to differentiate ICOs to mature cholangiocyte phenotype. CDM, cholangiocyte differentiation medium; CDM+FSK, CDM with Forskolin medium; M+C, Matrigel and collagen type I mixed hydrogel. B) Morphology of ICOs in CDM condition at different days. Scale bar = 100 µm. C,D) Gene expression analysis for cholangiocyte organoids in M+C hydrogel. Four independent donors for CDM conditions at five time points (day 0, 2, 4, 7, and 9). Results are shown as fold change relative to human liver tissue for adult stem cell and hepatocyte markers and human gallbladder for the cholangiocyte markers. Data are shown as box and whisker plots. Center line, median; box, interquartile; whiskers: minimum to maximum, show all points. Statistical differences between groups were tested using one‐way ANOVA followed by Dunn's test for multiple comparisons; *n* = 4, *p < 0.05, **p < 0.01.

Next, ICOs were mechanically dissociated into small fragments and resuspended in droplets of Matrigel mixed with collagen type I, and maintained in CDM with forskolin for 2 d to promote the regeneration of organoids, followed by culture in CDM to drive differentiation (**Figure** **1**A). After culturing in CDM, organoids became denser and formed thicker organoid walls indicating differentiation (Figure 1B). At the gene expression level, the stem/progenitor marker leucine rich repeat containing G protein‐coupled receptor 5 (*LGR5*) was downregulated in CDM at all time points suggesting decreased stemness (Figure 1C). Expression of the hepatocyte marker albumin (*ALB*) decreased and was lowest on day 4 in CDM whereas most of the cholangiocyte markers showed highest expression at day 4, with some exceptions that continued to increase until day 9. More specifically, the organoids expressed cholangiocyte markers (Figure 1D), including hepatocyte nuclear factor 1 beta (*HNF1β*), SRY‐box transcription factor 9 (*SOX9*), keratin 7 (*KRT7*), keratin 19 (*KRT19*), cystic fibrosis transmembrane conductance regulator (*CFTR*), and aquaporin 1 (*AQP1*) in improved CDM conditions. Interestingly, *HEY1*, solute carrier family 4 member 2 (*SLC4A2*), *SLC10A2*, *SSTR2*, and G protein‐coupled bile acid receptor 1 (*GPBAR1*) showed a trend of increased expression from day 4 to day 9. In addition, cholangiocyte markers Jagged canonical Notch ligand 1 (*JAG1*), *SPP1*, gamma‐glutamyl transferase 1 (*GGT1*), and *ABCB1* were significantly increased on day 7 compared to day 0. *SOX9* showed a decrease at day 4 compared to day 2, most likely due to the removal of forskolin in CDM, which can influence SOX9 by the cAMP/PKA pathway.^[^ ^15^ ^]^ Together, ICOs in expansion medium displayed higher expression of cholangiocyte markers (*KRT7* and *KRT19*) compared to human primary gallbladder cholangiocytes (threefold to fivefold change). However, the expression of functional cholangiocyte markers decreased. Maturation of ICOs in CDM resulted in an upregulation of *GGT1* and *ABCB1*. In addition, the cholangiocyte bile acid transport markers (*SLC4A4*, *SLC10A2*, *GPBAR1*) and *SSTR2* showed a trend toward upregulation under CDM conditions. These results indicate that ICOs display a more mature cholangiocyte phenotype after culture for CDM.

### 2.2 PIC Hydrogel Enhances ICO Differentiation into Mature Cholangiocyte

**Figure 2:**
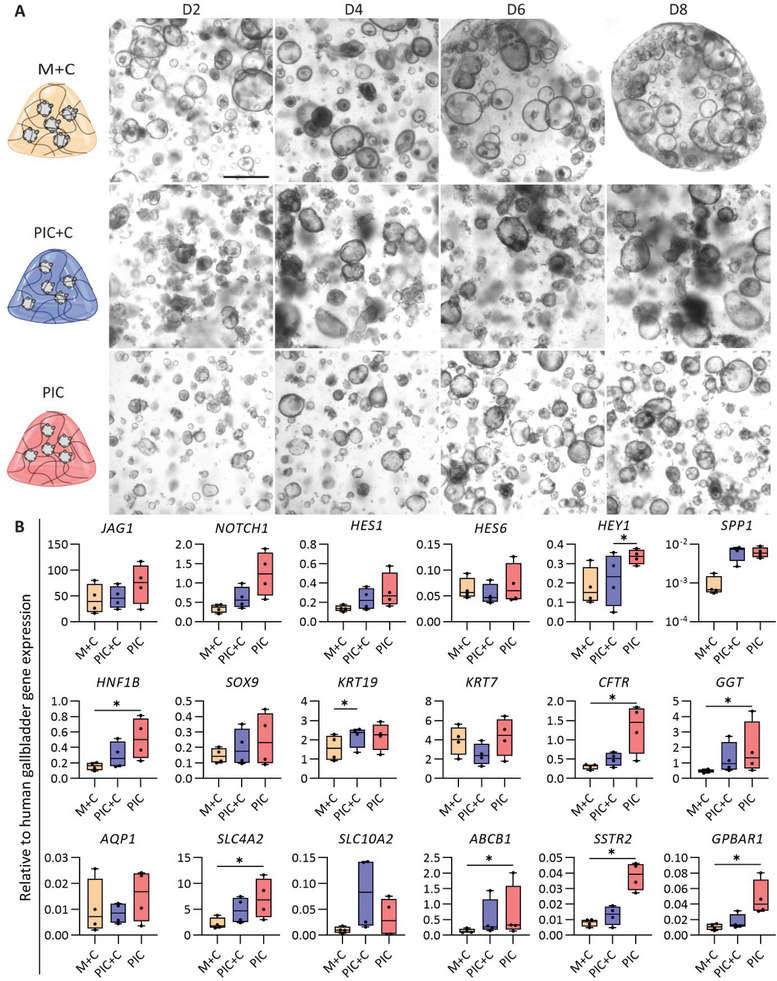
Generation of mature cholangiocytes from human intrahepatic cholangiocyte organoids (ICOs) in different hydrogels. A) Morphology of the mature cholangiocyte organoids in different hydrogels at four time points of differentiation (day 2, 4, 6, and 8). M+C, Matrigel/collagen type I mixed hydrogel; PIC+C, PIC/collagen I mixed hydrogel; PIC, PIC hydrogel. Scale bar = 500 µm. B) Gene expression analysis for cholangiocyte organoids in CDM conditions in different hydrogels after differentiation. Four independent donors in CDM conditions in three different hydrogels (M+C, PIC+C, and PIC hydrogel) at day 7. Results are shown as fold change relative to human gallbladder. Data are shown as box and whisker plots. Center line, median; box, interquartile; whiskers: minimum to maximum, shows all points. Statistical differences between the groups were tested using one‐way ANOVA followed by Dunn's test for multiple comparisons; *n* = 4, *p < 0.05.

As shown in Figure 1, differentiation of ICOs toward the cholangiocyte lineage can be enhanced in Matrigel and collagen type I mixed hydrogel with novel medium adaptations. In order to drive increased biliary function and circumvent the batch‐to‐batch variation of Matrigel, as well as avoid the presence of proliferation‐inducing ECM components, such as laminins, we tested ICO differentiation in a fully defined, synthetic PIC hydrogel, in the presence of CDM. Previously, we used PIC to maintain ICO cultures and provided extensive rheological analysis of the hydrogel.^[^ ^6^ ^]^ Here, we compared the PIC with the “standard” natural hydrogel (Matrigel mixed with collagen I, M+C) as a control, as well as the mixed PIC and collagen type I (PIC+C) hydrogel for ICO differentiation into the cholangiocyte lineage. Bright field analysis indicated that the organoids were smaller and denser in the PIC+C and PIC hydrogel compared to M+C hydrogel (**Figure** **2**A) following differentiation in CDM. Furthermore, the proliferation marker Ki67 showed decreased expression compared to liver biopsy sample was absent at the protein level. These results indicate that the organoids exit their proliferative state as they mature into cholangiocytes (Figure S2, Supporting Information). Gene expression analysis indicated that *KRT19* showed increased expression in PIC+C hydrogels compared to M+C hydrogel. Interestingly, we observed that seven cholangiocyte markers (namely, *HNF1β*, *CFTR*, *GGT1*, *SLC4A2*, *ABCB1*, *SSTR2*, *GPBAR1*) showed increased expression, and eight cholangiocyte markers (*JAG1*, *NOTCH1*, *HES1*, *HEY1*, *SPP1*, *SOX9*, *KRT19*, *SLC10A2*) displayed a trend toward upregulation. Only three genes (*HES6*, *KRT7*, *AQP1*) maintained the same levels of expression when compared to M+C hydrogel control. More importantly, only synthetic PIC hydrogel showed an increased expression of the cholangiocyte transporters (namely, *CFTR*, *GGT1*, *SLC4A2*, *ABCB1*, *SSTR2*, *GPBAR1*) compared to M+C hydrogel control (Figure 2B). In summary, gene expression analysis showed that differentiation of ICOs in PIC hydrogel increased the expression of mature cholangiocyte genes and outperformed the PIC+C as well as the M+C hydrogel control.

**Figure 3:**
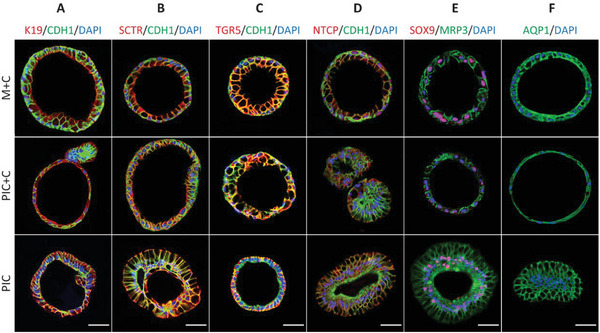
Immunofluorescence analysis of the key cholangiocyte markers in organoids cultured in three different hydrogels (M+C, Matrigel/collagen I; PIC+C, PIC/collagen I; PIC). A) Cytoskeleton, epithelial and junction markers [keratin (K) 19, cadherin 1 (CDH1) and tight junction protein 1 (ZO1)]. B–E) Regulator of bile acid markers [secretin receptor (SCTR), G protein‐coupled bile acid receptor 1 (GPBAR1, also known as TGR5), Na^+^‐taurocholate cotransporting polypeptide (NTCP), multidrug resistance protein 3 (MRP3)]. (E) Transcription factor marker [SRY‐box transcription factor (SOX9)] and the water channel marker (F) aquaporin 1 (AQP1). Scale bar = 50 µm.

**Figure 4:**
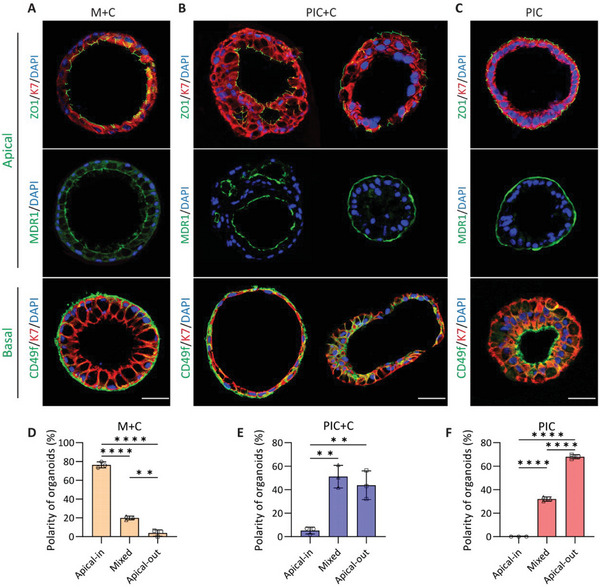
Immunofluorescence analysis of the cholangiocyte organoids polarity in the three different hydrogels (M+C, Matrigel/collagen I; PIC+C, PIC/collagen I; PIC), and quantified for percentage of apical‐inside, mixed or apical‐out organoids. A) Organoids cultured in M+C hydrogel showed apicobasal polarity (apical side located at the inside of organoid), as indicated by apical markers (ZO1 and MDR1) and the basolateral marker (CD49f / integrin α 6), and quantified 176 organoids for polarity (D). B) Organoids in PIC+C hydrogel showed two kinds of apicobasal polarity, the apical‐out polarization and the normal apical‐in organoid orientation, for which 262 organoids were quantified for polarity (E). C) However, PIC cultured organoid showed apicobasal polarity, opposite to M+C cultures (apical‐inside organoid), for which 161 organoids were quantified for polarity (F). Scale bar = 50 µm, and data are shown as mean ± SD of three independent donors for each group. Statistical differences between the groups were tested using one‐way ANOVA followed by Tukey's test for multiple comparisons; *n* = 3, **p < 0.01, ****p < 0.0001.

Next, we performed immunostainings of differentiated ICOs in the different hydrogels (M+C, PIC+C, and PIC) to determine the presence of key cholangiocyte markers on protein level. This revealed the presence of epithelial markers of tight junction protein 1 (TJP1, also known as ZO1) and E‐cadherin 1 (CDH1), as well as cholangiocyte cytoskeleton markers, K7 and K19 (**Figures** **3**A and **4**). Furthermore, the organoids expressed key cholangiocyte markers, including regulators of bile acid modification and transportation markers, secretin receptor (SCTR), GPBAR1 (also known as TGR5), sodium/bile acid cotransporter, also known as the Na^+^‐taurocholate cotransporting polypeptide (NTCP), ATP binding cassette subfamily C member 3 (MRP3, encoded by *ABCC3*) in Figure 3B–E, biliary transcription marker SOX9 (Figure 3E) and the water channel aquaporin‐1 (AQP1) showed in Figure 3F. In addition, organoids in the three different hydrogels were positive for ATP binding cassette subfamily B member 1 (MDR1, encoded by *ABCB1*) in Figure 4. Together, these markers confirmed that the organoids acquired a mature cholangiocyte phenotype in three different hydrogels.

Next, we analyzed the epithelial polarity of cholangiocyte organoids through the localization of cholangiocyte markers by immunofluorescent analysis. As shown in Figure 4, organoids in three different hydrogels showed localized expression of ZO1, MDR1 and Integrin alpha 6 (CD49f) suggesting that the organoids acquired an apicobasal polarity. The organoids in M+C hydrogel culture showed mostly apicobasal polarity, with the apical side facing the lumen in approximately 76% of the organoids. However, the organoids in PIC hydrogel displayed mostly an inverted polarity with the apical membrane located at the outside in approximately 68% of the organoids. Interestingly, we observed that the organoids cultured in PIC+C hydrogel show mainly two kinds of apicobasal polarity: one with the apical structure located at the luminal side in approximately 5% of the organoids, and the other with the apical structure located at the outside in approximately 44% of the organoids (Figure 4).

### 2.3 Functional Characterization of Cholangiocyte Organoids

**Figure 5:**
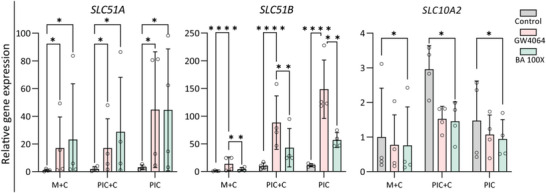
Functional FXR signaling in cholangiocyte differentiated organoids in three different hydrogels (M+C, Matrigel/collagen I; PIC+C, PIC/collagen I; PIC). Gene expression analysis showing the activation of FXR signaling by downstream upregulated genes, *SLC51A*, *SLC51B*, downregulated genes *SLC10A2* in DMSO (Control), FXR agonist (GW4064) and cholestatic condition (BA 100×) group. Data are shown as mean ± SD of four independent donors for each group. Statistical differences between the groups were tested using two‐way ANOVA; *n* = 4, *p < 0.05, **p < 0.01, ****p < 0.0001.

In the liver, one of the major physiological functions of cholangiocyte is their modification of bile. To examine the functionality of mature cholangiocytes in M+C, PIC+C, and PIC hydrogels in vitro, we tested activation of the Farnesoid X receptor (FXR), one of the most important bile acids activated nuclear receptor controlling bile excretion to avoid excessive bile accumulation leading to cholestasis. Cholangiocytes possess specific bile acid transporters including ASBT (encoded by *SCL10A2*), an apical transporter posed to take up biliary bile acids, and organic solute transporter α/β (encoded by *SCL51A* and *SLC51B*, respectively), which combined are responsible for a basolateral efflux of bile acids. After incubation with GW4064, an FXR agonist, a decreased expression of *SLC10A2* and increased expression of *SLC51A/B* was observed in line with the nuclear receptor activation. Furthermore, the organoids showed similar expression pattern trends in response to high concentrations of bile acids mimicking cholestatic conditions in all three different hydrogels (**Figure** **5**). Taken together, these data indicate that the mature cholangiocyte organoids are able to perform FXR‐mediated bile homeostasis, which can be used to mimic cholestasis.

### 2.4 Mature Cholangiocyte Organoids to Model Biliary Pro‐Fibrotic Response

**Figure 6:**
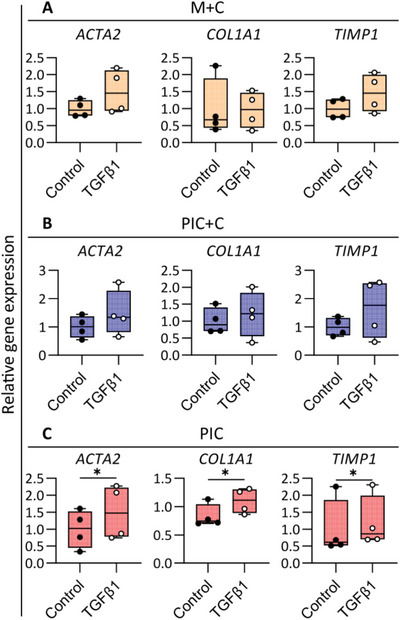
Modeling pro‐fibrotic responses in cholangiocyte organoids in three different hydrogels (M+C, Matrigel/collagen I; PIC+C, PIC/collagen I; PIC). Gene expression analysis showing the activation of fibrosis related genes *ACTA2*, extracellular matrix gene *COL1A1*, and metallopeptidase inhibitor gene *TIMP1* in the TGFβ1 treated group versus unstimulated conditions (Control). Data are shown as mean ± SD of four independent donors for each group. Statistical differences between the groups were tested using paired t‐test (one‐tailed); *n* = 4, * *p* < 0.05.

With the aim to extend and expand on our previous efforts highlighting the translational relevance of cholangiocyte organoids cultured in our animal‐free culture system, we investigated the organoid's ability to model TGFβ‐induced biliary fibrosis. During fibrosis, (prolonged) activation of TGFβ signaling leads to matrix accumulation and, subsequently, organ dysfunction.^[^ ^16^ ^]^ After 6 days of culturing ICOs in three different hydrogels (M+C, PIC+C, and PIC) in CDM, the organoids were incubated in the absence and presence of TGFβ (25 ng mL^−1^) for 48 h, after which fibrosis related gene expressions were evaluated. As shown in **Figure** **6**, actin alpha 2 (*ACTA2*), collagen type I alpha 1 chain (*COL1A1*), and tissue inhibitor of metalloproteinases 1 (*TIMP1*) showed a trend toward upregulation in the M+C and PIC+C hydrogels after TGFβ pathway activation. Interestingly, these three genes (*ACTA2, COL1A1*, and *TIMP1*) were all increased after TGFβ activation in PIC hydrogel (Figure 6). These results suggest that the organoids are potentially suitable for biliary fibrosis modeling.

## 3 Discussion

ICOs derived from primary tissue of the human liver are a powerful technology for disease modeling, tissue engineering and regenerative medicine applications. Compared to cell lines, biopsy‐derived organoids exhibit a high inter‐donor variability, which is inherent to diversity in genetic background. Previously, we reported that human and murine ICOs can be differentiated into mature cholangiocytes in an animal‐derived hydrogel (namely, the combination of Matrigel and collagen I) using a defined differentiation media.^[^ ^2^, ^4^ ^]^ However, despite improving the overall differentiation into the cholangiocyte lineage, these culture conditions had several limitations due to the poorly defined and variable composition of Matrigel. This variability impeded the complete maturation of cells into cholangiocytes because of the presence of proliferative cues within the matrix. With the development of chemically‐defined hydrogels for liver organoid expansion and differentiation into the hepatocyte lineage (e.g., PEG and PIC), a new opportunity arose for cholangiocyte differentiation.^[^ ^6^, ^8^, ^9^ ^]^ Previously, we found PIC hydrogel alone was not sufficient to support ICOs growth, but PIC combined with lamine‐111 promote organoid formation and proliferation.^[^ ^6^ ^]^ However, these bioactive motifs including laminin are important for organoids growth, but may hamper their differentiation. Whether the PIC hydrogel alone could support ICOs differentiation is the issue tackled in this study. Herein, we first optimized the CDM media conditions and subsequently applied a synthetic PIC hydrogel to differentiate ICOs to a more mature cholangiocyte phenotype. Indeed, under these conditions the organoids express various cholangiocyte markers, and potentially mimic cholangiocyte functions to a greater extent than their Matrigel cultures counterparts.

The generation of functional cholangiocytes from various cell sources has been reviewed elsewhere.^[^ ^17^ ^]^ Of importance in cholangiocyte biology are the TGFβ‐ and Notch signaling pathways, known to be essential for maturation and morphogenesis of bile duct development in vivo.^[^ ^18^ ^]^ Rizwan et al. reported that Jag1 combined with hyaluronan hydrogel activates hepatoblast differentiation into cholangiocytes.^[^ ^19^ ^]^ In addition, it has been confirmed that VPA treatment induces hepatoblast differentiation into cholangiocyte‐like cells by activating Notch signaling.^[^ ^20^ ^]^ Therefore, one method to improve cholangiocyte differentiation would be via activating the Notch signaling pathway to drive differentiation of cholangiocytes from progenitor/stem cells.^[^ ^8^, ^21^ ^]^ Furthermore, histone deacetylase 1 (HDAC1) is known to regulate hepatocyte differentiation from liver progenitor cells.^[^ ^22^ ^]^ Previous studies have reported that both valproic acid and sodium butyrate, which can inhibit histone deacetylase activate the Notch pathway.^[^ ^23^ ^]^ When ICOs were stimulated by sodium butyrate, the expression of *ALB* and *AFP* were downregulated, indicating that sodium butyrate can drive the ICOs away from the hepatocyte lineage.^[^ ^24^ ^]^ With the condition where CDM was supplemented with VPA, the expression of *HEY1*, *SPP1*, *ABCB1*, *SLC10A2*, and *SSTR2* was increased, which indicates that further maturation toward the cholangiocyte lineage was successful in ICOs. In vivo, intrahepatic cholangiocytes display functional and morphological heterogeneity. Compared to small cholangiocytes, large cholangiocytes are involved in secretin and somatostatin signaling via their corresponding receptors.^[^ ^25^ ^]^ In addition, only large cholangiocytes express CFTR and AE2 and are responsible for bile modification through the activation of a cAMP‐dependent pathway.^[^ ^26^ ^]^ Importantly, our research revealed that ICOs‐derived cholangiocytes showed gene expression of *SSTR2* and *SLC4A2*, and positive staining for SCTR. These results support the influence of Notch signaling on the generation of mature cholangiocytes from ICOs, arguing for a large cholangiocyte phenotype.

Several studies reported that (liver‐)specific ECM components derived from human and porcine liver can support ICO proliferation and differentiation in vitro.^[^ ^27^ ^]^ Furthermore, liver‐specific ECM, derived from decellularized porcine livers, maintain a complex biliary network^[^ ^28^ ^]^ suitable to support HepaRG cells to form hepatic tissue for drug screening.^[^ ^29^ ^]^ More specifically, Willemse et al., showed that human or porcine liver‐derived ECM could support ICO proliferation, although this ECM hydrogel did not affect hepatobiliary marker expression during expansion. They concluded that the culture medium is more important to obtain the cholangiocyte phenotype than the ECM components present during culturing.^[^ ^27a^ ^]^ However, PIC cultured salivary organoids were enriched more differentiation correlated genes compared to Matrigel cultured.^[^ ^12^ ^]^ Based upon these research papers, we expected a switch in cholangiocyte phenotype when ICO culture conditions were changed from the biological hydrogel to a chemically defined hydrogel. Indeed, the use of PIC hydrogel improved expression of cholangiocyte markers, including *HNF1β*, *CFTR*, *GGT*, *SLC4A2*, *ABCB1*, *SSTR2*, and *GPBAR1*. Moreover, the immunofluorescence analysis indicated that the organoids maintain the expression of cholangiocyte‐specific proteins. These results suggest that the PIC hydrogel favors maturation of ICO‐derived cholangiocytes. With regards to functionality, the cholangiocyte organoids acquired several cholangiocyte specific functional markers when cultured in PIC, including the FXR signaling pathway that regulates bile acid homeostasis.^[^ ^30^ ^]^ Overall, the ICO‐derived cholangiocytes achieved a higher maturation level in PIC compared to the previously used natural hydrogel and demonstrate important cholangiocyte functions.^[^ ^2^ ^]^

With regards to polarity, the organoids differentiated in the PIC hydrogel displayed a reversed polarity compared to organoids differentiated in Matrigel, where transporters are present in the apical membrane and localized to the outside of the organoids, rather than lining the lumen within the organoids. This contradicts to the inside presence of apical markers when cultured in Matrigel/collagen I hydrogel. In vivo, cholangiocytes have transporters in an apicobasal configuration (OSTα/β, ASBT, and MDR3) which perform key physiological functions in maintaining bile acids homeostasis in the liver.^[^ ^31^ ^]^ Such configuration could be of interest when considering ICO‐derived cholangiocytes for disease modeling. Of note, a similar polarity has been reported for cholangiocyte organoids cultured in hyaluronan^[^ ^8^ ^]^ or cellulose nanofibril hydrogel,^[^ ^32^ ^]^ and suspension culture for intestinal organoids^[^ ^33^ ^]^ or pancreatic duct organoids.^[^ ^34^ ^]^ The apical‐out organoids can be used in specific experiments where this orientation is preferred. For instance, enteroids and gastric organoids with an apical‐out orientation could efficiently absorb fatty acids and can be used for apical infection studies of pathogens.^[^ ^33^, ^35^ ^]^ Moreover, we found the ICOs in mixed hydrogel (PIC with Collagen type I) showed two different phenotypes of polarity. The most pronounced difference between organoids cultured in hydrogel was the polarity change in PIC only. Thus, our results provide an important clue for the cell‐matrix influence on the organoids polarity reversal.^[^ ^36^ ^]^ Given the unexpected location of polarity markers, in accordance with the ECM plays a crucial role on the epithelial cell polarity and morphogenesis.^[^ ^37^ ^]^ Integrins are transmembrane receptors that mediate cellular adhesion and migration to neighboring cells or to ECM components.^[^ ^36^ ^]^ Due to the shortage of integrin ligands (e.g., fibronectin, osteopontin, vitronectin, and fibrinogen) in the synthetic PIC hydrogel, the organoids showed an apical‐out phenotype in this matrix.

To date, organoids have been used in a variety of cholangiopathy studies.^[^ ^38^ ^]^ We showed that ICOs cultured in PIC hydrogel could potentially maintain bile acid homeostasis and be used to study drug‐induced cholestatic conditions, as previously described.^[^ ^1a^ ^]^ For further exploration of the PIC hydrogel in ICO applications, we tested the system for TGFβ signaling induced pro‐fibrotic phenotype. Our results showed that the organoids acquired a pro‐fibrotic phenotype after TGFβ stimulation based on gene expression, consistent with reported findings on ECM deposition.^[^ ^39^ ^]^

## 4 Conclusion

In conclusion, animal‐derived hydrogels hamper the clinical application of cholangiocyte organoids for tissue engineering and regenerative medicine. PIC as a chemically defined scaffold could replace these hydrogels for organoid culture in a more standardized fashion in in vitro modeling or even clinical applications. In this work, we generated mature cholangiocyte organoids derived from ICOs in a fully defined matrix (PIC hydrogel) and optimized culture medium. Using this PIC hydrogel, we were able to support a more mature cholangiocyte phenotype in contrast to organoids cultured in the animal‐derived (mixed) hydrogel (Matrigel and collagen I) used previously. The PIC hydrogel supports maturation of organoid derived cholangiocytes which acquired an apical‐out polarity. The cholangiocyte organoids can be used for cholangiocyte specific in vitro modeling, such as pathogen infections and/or studying apical transport processes.

## 5 Experimental Section

### Hydrogel Preparation

Mixed Matrigel (Corning, New York, NY) and rat‐tail type I collagen (1.2 mg mL^−1^; Merck Millipore) at a ratio of 2:3 hydrogel (Matrigel/collagen I, M+C). Noviogel (PIC, 1k‐PIC‐P) was purchased from Sopachem (The Netherlands) and prepared at a concentration of 1 mg mL^−1^ PIC hydrogel. PIC+C (PIC/collagen I) hydrogel was prepared via PIC (2 mg mL^−1^) and collagen I (2 mg mL^−1^) hydrogel mixed at a ratio of 1:1.

### ICO Establishment and Expansion

Liver biopsies from healthy livers were obtained during liver transplantation at the Erasmus Medical Center in Rotterdam, approved by the Medical Ethical Council (MEC‐2014‐060). The human ICOs were established and cultured as previously described.^[^ ^40^ ^]^ Briefly, the human liver biopsies were minced into small fragments and enzymatically digested by 0.125 mg mL^−1^ type II collagenase (Gibco, Thermo Fisher Scientific, Waltham, MA) and 0.125 mg mL^−1^ dispase (Gibco) in DMEM GlutaMAX medium (Gibco) supplemented with 0.1 mg mL^−1^ DNase I (Roche, Basel, Switzerland), 1% (v/v) fetal calf serum (FCS; Gibco), and 1% (v/v) penicillin/streptomycin (P/S; Gibco) in 37 °C shaking water bath. The supernatant was collected, and fresh enzyme‐supplemented medium was added every 10–15 min for three times for tissue digestion. Single cells were washed in cold DMEM GlutaMAX medium supplemented with 1% (v/v) FCS and 1% (v/v) P/S and centrifuged at 400 *g* for 5 min. The cells were resuspended in cold Matrigel droplets and expansion medium (EM) was added after gelation. The EM was consisted of Advanced DMEM/F12 medium (Gibco) supplemented with 1% (v/v) P/S, 10 × 10^−3^ m HEPES (Gibco), 1% (v/v) GlutaMax (Gibco), 10% (v/v) Rspondin‐1 conditioned medium (the Rspon1‐Fc‐expressing cell line was a kind gift from Calvin J. Kuo), 2% (v/v) B27 supplement without vitamin A (Invitrogen, Carlsbad, CA), 1% (v/v) N2 supplement (Invitrogen), 1.25 × 10^−3^ m N‐acetylcysteine (NAC; Sigma‐Aldrich, St. Louis, MO), 10 × 10^−3^ m nicotinamide (Sigma‐Aldrich), 10 × 10^−9^ m recombinant human (Leu15)‐gastrin I (GAS; Tocris Bioscience, Bristol, UK), 50 ng mL^−1^ epidermal growth factor (EGF; Peprotech, Rocky Hill, NJ), 25 ng mL^−1^ hepatocyte growth factor (HGF; Peprotech), 100 ng mL^−1^ fibroblast growth factor 10 (FGF10; Peprotech), 10 × 10^−6^ m Forskolin (FSK; Tocris Bioscience), and 5 × 10^−6^ m A8301 (transforming growth factor β inhibitor; Tocris Bioscience). Organoids were passaged at 1:3–1:4 ratio each week and medium was refreshed every 2–3 d.

### ICO Differentiation to Mature Cholangiocyte Organoids

The activity of SB (Sigma) and VPA (Sigma) was examined using ICOs differentiation into mature cholangiocyte organoids. ICOs were mechanically passaged at 1:2 ratio and seeded in fresh hydrogel (M+C; 40% Matrigel and 60% 1.2 mg mL^−1^ rat‐tail type I collagen (Merck Millipore)). After hydrogel gelation, ≈30 min, the CDM was added. The CDM was modified as follows: Advanced DMEM/F12 medium supplemented with 1% (v/v) P/S, 1% (v/v) GlutaMax, 10 × 10^−3^ m HEPES, 1% (v/v) ITS Premix (5 µg mL^−1^ insulin, 5 µg mL^−1^ transferrin, and 5 µg mL^−1^ selenous acid; Corning), 2% (v/v) B27 supplement without vitamin A, 1.25 × 10^−3^ m NAC, 10 × 10^−9^ m GAS, 50 ng mL^−1^ EGF, 25 ng mL^−1^ HGF, 100 ng mL^−1^ FGF10, 10 × 10^−6^ m FSK, 1 × 10^−3^ m sodium butyrate (Sigma), and 1 × 10^−3^ m valproic acid (Sigma). After two days culture, the condition medium was removed FSK and changed every two days from day 2 to day 9.

### RNA Isolation and RT‐qPCR

The TRIzol Reagent (Invitrogen) was used to isolate total RNA from liver biopsy, gallbladder and organoids following the manufacturer's instructions. RNA quality and quantity were measured with the ND‐1000 spectrophotometer (NanoDrop, Thermo Fisher Scientific). cDNA synthesis was performed using the iScript cDNA synthesis kit (Bio‐Rad, Hercules, California, USA), and qPCR was performed using the SYBR Green method (Bio‐Rad) according to the manufacturer's instructions. Hypoxanthine phosphoribosyltransferase 1 (*HPRT1*), ribosomal protein S5 (*RPS5*), and tyrosine 3‐monooxygenase/tryptophan 5‐monooxygenase activation protein zeta (YWHAZ) were selected as stably expressed reference genes, and their average expression was used for normalization. Details of all primers are described in Table S1 (Supporting Information).

### Immunofluorescence Analysis

Organoids were fixed with 4% (v/v) paraformaldehyde solution for 1 h at room temperature. Fixed samples were dehydrated in 70% (v/v) ethanol and embedded in paraffin. The deparaffinized and rehydrated sections were incubated at 98 °C 30 min in Tris‐EDTA (PH 9.0) for antigen retrieval. Nonspecific antibody binding was avoided by adding 10% (v/v) goat serum in PBS for 1 h and primary antibodies were incubated overnight at 4 °C. After being washed in PBS with 0.1% Tween (Sigma), slides were incubated with secondary antibodies and nuclei were stained with DAPI (1:1000; Sigma‐Aldrich) for 1 h. Finally, slides were washed with PBS and mounted with Prolong Diamond Antifade Mounting Medium (Invitrogen). Images were acquired using a Leica TCS SP8 X imaging system. Details of all antibodies are described in Table S2 (Supporting Information).

### FXR Signaling Pathway and Bile Acids Homeostasis Assay

For the bile acid homeostasis assay, organoids were pretreated with DMSO (Control), 10 × 10^−6^ m GW4064 (an agonist of FXR; Sigma‐Aldrich), or a hundred‐fold concentrated bile acid cocktail to mimic cholestatic condition (BA 100× cocktail consisting of 132.0 × 10^−6^ m glycochenodeoxycholate, 40.0 × 10^−6^ m deoxycholic acid, 39.0 × 10^−6^ m chenodeoxycholic acid, 35.0 × 10^−6^ m glycocholic acid, 31.0 × 10^−6^ m glycodeoxycholic acid; all bile acid were purchased from Sigma‐Aldrich) for 48 h. RNA was isolated and used to evaluate the bile acid transporter gene expression.

### Model Phenotype of Biliary Pro‐Fibrotic Response

Organoids were pretreated with culture medium (CDM, Control), culture medium with 25 ng mL^−1^ TGFβ (Sigma‐Aldrich) for 48 h. RNA was isolated and used to evaluate the biliary fibrosis gene expression.

### Statistical Analysis

Statistical analysis was performed using GraphPad Prism 9 (GraphPad Software). Data are presented as box and whisker plots (minimum to maximum, Figures 1C,D and 2B), or the mean ± standard deviation (Figures 4, 5, and 6). Statistical differences between the groups were tested using one‐way ANOVA followed by Dunn's test (Figures 1C,D and 2B) or Tukey's test (Figure 4D–F), two‐way ANOVA (Figure 5) or paired Student's t‐test (Figure 6). Results were considered statistically significant when *p < 0.05, and details are described in the figure legends.

## Conflict of Interest

The authors declare no conflict of interest.

## Author Contributions

Z.W. and S.Y. contributed equally to this work. Z.W., S.Y., R.M., and B.S. conceived the project and designed the experiment. Z.W. and S.Y. performed the experimental work and data analysis. Z.W. provided the original draft preparation. R.M. and B.S. guided the project and critically revised the manuscript. K.S. and L.L. provided suggestions and critically revised the manuscript. All authors read and approved the final submitted manuscript.

## Supporting information

Supporting Information

## Data Availability

The data that support the findings of this study are available in the Supporting Information of this article.
