# Supplementary material for: Chemically Defined Organoid Culture System for Cholangiocyte Differentiation
Source: Adv Healthc Mater. 2024 Jul 23;13(30):2401511. doi: 10.1002/adhm.202401511 (PMC11616262; doi:10.1002/adhm.202401511)
Supplement: Supplementary file 1 — Supporting Information [file ADHM-13-0-s001.docx]

**Chemically Defined Organoid Culture System for Cholangiocyte Differentiation**

*Zhenguo Wang^#^, Shicheng Ye^#^, Luc J.W. van der Laan, Kerstin Schneeberger, Rosalinde Masereeuw^*^, Bart Spee^*^*

**Supporting information**


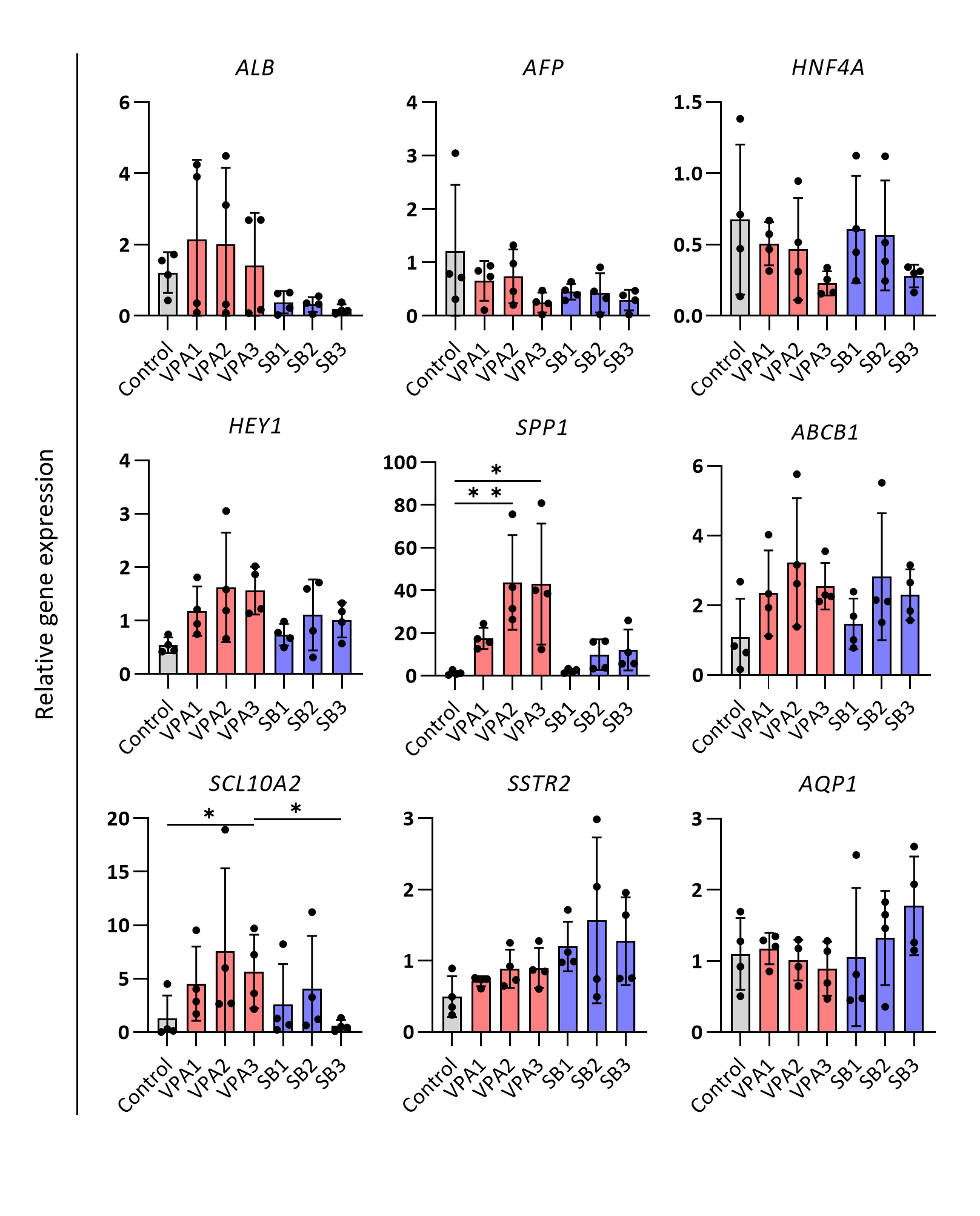


**Figure S1.** Gene expression analysis showing key hepatocyte- and cholangiocyte-markers. ICOs were treated with different concentrations of valproic acid (VPA; 1, 2, 4 mM), and sodium butyrate (SB; 1, 2, 4 mM). Data are shown as mean ± SD of four independent experiments for each group. Statistical differences between groups were using one-way ANOVA followed by Dunn’s test for multiple comparisons; n = 4, **P* < 0.05, ***P* < 0.01.

**
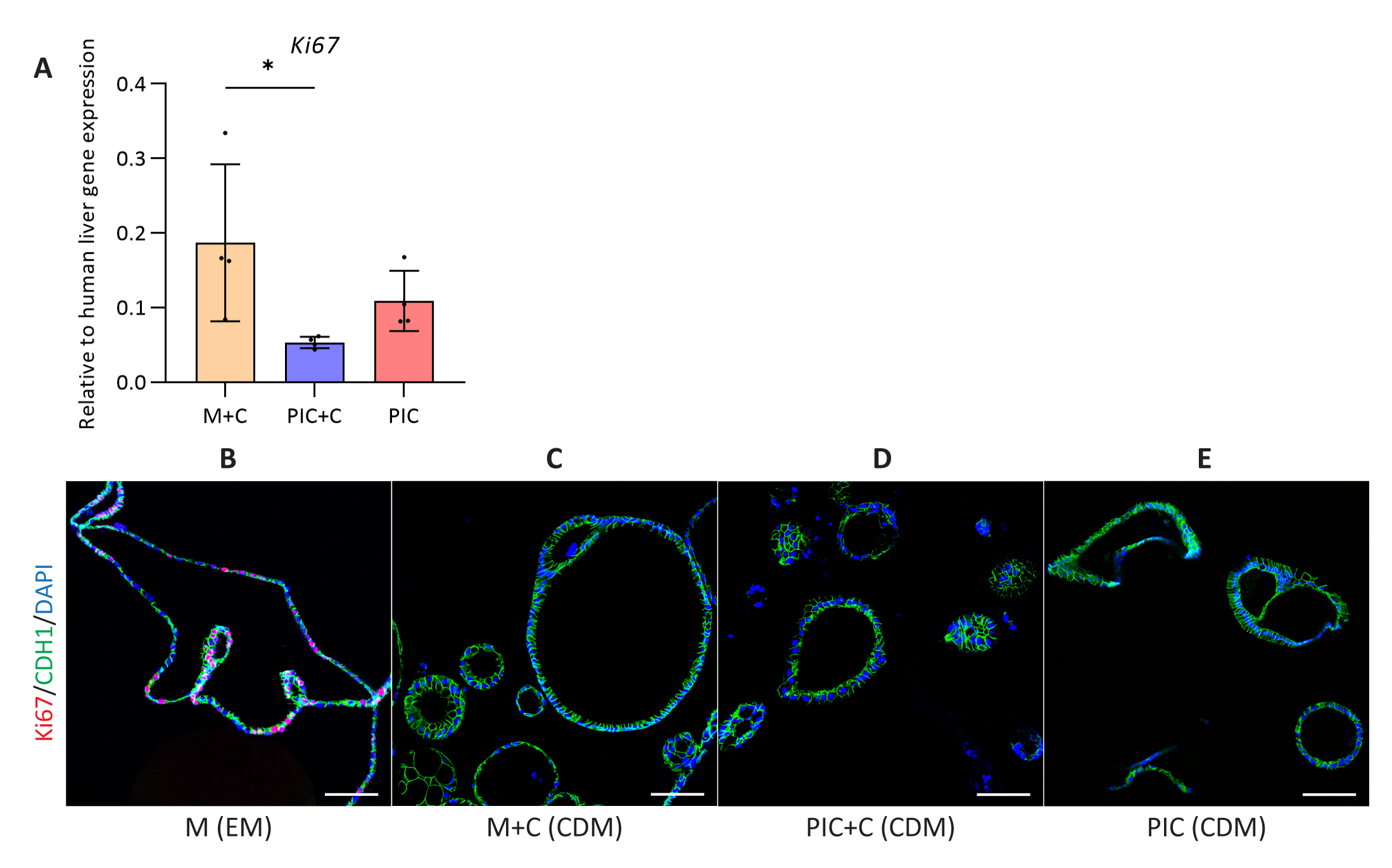
**

**Figure S2.** Analysis of ICOs derived mature cholangiocytes proliferation ability in different hydrogel.

(A) Gene expression analysis for cholangiocyte organoids in CDM (cholangiocytes differentiation medium) conditions in different hydrogels after differentiation. M+C, Matrigel/collagen type Ⅰ mixed hydrogel; PIC+C, PIC/collagen Ⅰ mixed hydrogel; PIC, PIC hydrogel. Results are shown as fold change relative to human liver tissue for proliferation marker *Ki67*. Data are shown as mean ± SD of four independent experiments for each group. Statistical differences between groups were using one-way ANOVA followed by Dunn’s test for multiple comparisons; n = 4, **P* < 0.05.

(B-E) Immunofluorescence analysis of the proliferation marker in organoids cultured in the three different hydrogels after differentiation. (B) ICOs cultured in Matrigel (M) in expansion medium (EM) showed proliferation marker Ki67. The proliferation ability was absent in three different hydrogel after differentiation (C-E). Epithelial marker [cadherin 1 (CDH1)], scale bar = 100 µm.

| **Table S1.** List of primers used in RT-qPCR analysis | | |
| --- | --- | --- |
| *Gene* | *Forward primer* | *Reverse primer* |
| *HPRT1* | TATTGTAATGACCAGTCAACAG | GGTCCTTTTCACCAGCAAG |
| *RPS5* | TGCAGGATTACATTGCAGTG | CATCATGGAGTTAGTGAGGC |
| *YWHAZ* | ACTTTTGGTACATTGTGGCTTCAA | CCGCCAGGACAAACCAGTAT |
| *Ki67* | GCTACTCCAAAGAAGCCTGTG | AAGTTGTTGAGCACTCTGTAGG |
| *LGR5* | GCAGTGTTCACCTTCCC | GGTCCACACTCCAATTCTG |
| *ALB* | GTTCGTTACACCAAGAAAGTACC | GACCACGGATAGATAGTCTTCTG |
| *AFP* | GATGAAACATATGTCCCTCCTG | ATGAGAAACTCTTGCTTCATCG |
| *HNF4A* | GTACTCCTGCAGATTTAGCC | CTGTCCTCATAGCTTGACCT |
| *JAG1* | CTTGTGTAAACGCCAAATCC | CCATTAACCAAATCCCGACAG |
| *HEY1* | CGAGGTGGAGAAGGAGAGTG | TCGGCGCTTCTCAATTATTC |
| *HES1* | ACGTGCGAGGGCGTTAATAC | ATTGATCTGGGTCATGCAGTTG |
| *HNF1β* | ATGATCAAGGGTTACATGCAG | GTCTGGTTGAATTGTCGGAG |
| *SOX9* | CAAGCTCTGGAGACTTCTGAACG | CCGTTCTTCACCGACTTCCT |
| *SPP1* | TGTGCCATACCAGTTAAACAG | ATTCTGCTTCTGAGATGGGTC |
| *KRT7* | GGACATCGAGATCGCCACCT | ACCGCCACTGCTACTGCCA |
| *KRT19* | CTTCCGAACCAAGTTTGAGAC | AGCGTACTGATTTCCTCCTC |
| *CFTR* | TTCTGGGAGGAGGGATTTGG | GTGAGAAATTACTGAAGAAGAGGC |
| *GGT1* | CCTCAAAGGGTACAACTTCTC | TTGTAGTAGGAGATCGGGTG |
| *AQP1* | CACCTCCTGGCTATTGACTACAC | ATCCAGTGGTTGCTGAAGTTGTG |
| *SLC4A2* | ACTACCTGAGTGACTTCCGA | TGCTACAGAACGAGAAGAAGG |
| *SLC10A2* | CTGTGCCTCCTTATCTATACCA | AGAGAAACCAGAGATGTACCT |
| *ABCB1* | AATGATGCTGCTCAAGTTAAAGGG | TCAGTAGCGATCTTCCCAGAACC |
| *SSTR2* | GCAGTCCTCACATTCATCT | TGGTCTTCATCTTGGCATAG |
| *GPBAR1* | CATTGCCCACATTGCCAG | GAGCCAAGTAGACGAGGAG |
| *SLC51A* | TTGTTCGCCTCCCTATTCC | TTGTGGTCTTTCCTTCGGT |
| *SLC51B* | TGTGGTGGTCATTATAAGCATGG | TCTTAGGTTGTTTAGGCTGTTGTG |
| *ACTA2* | GTGTTGCCCCTGAAGAGCAT | GCTGGGACATTGAAAGTCTCA |
| *COL1A1* | TCCAACGAGATCGAGATCC | AAGCCGAATTCCTGGTCT |
| *TIMP1* | CTTCTGGCATCCTGTTGTTG | GGTATAAGGTGGTCTGGTTG |

| **Table S2.** List of antibodies used in immunofluorescent analysis | | | | |
| --- | --- | --- | --- | --- |
| *Antibody* | *Species* | *Supplier* | *Cat. No* | *Dilution* |
| Ki67 | Rabbit | Thermo Fisher | RM-9106-S | 1:100 |
| ZO1 | Rabbit | Invitrogen | 40-2300 | 1:250 |
| K7 | Mouse | Dako | M7018 | 1:100 |
| E-cadherin | Mouse | BD Bioscience | 610181 | 1:200 |
| K19 | Rabbit | Abcam | ab76539 | 1:150 |
| SOX9 | Rabbit | Abcam | ab185966 | 1:200 |
| MRP3 | Mouse | Abcam | ab3375 | 1:100 |
| HNF1β | Mouse | Atlas Antibodies | AMAb90733 | 1:200 |
| MDR1 | Rabbit | Novus Biologicals | NBP1-90291 | 1:200 |
| SCTR | Rabbit | Atlas Antibodies | HPA007269 | 1:250 |
| GPBAR1 | Rabbit | Atlas Antibodies | HPA062890 | 1:200 |
| ITGA6 | Rabbit | Novus Biologicals | NBP1-85747 | 1:200 |
| NTCP | Rabbit | Novus Biologicals | NBP1-60109 | 1:200 |
| Anti-mouse Alexa 488 | Goat | Thermo Fisher | A-11029 | 1:300 |
| Anti-Rabbit Alexa 568 | Goat | Thermo Fisher | A-11036 | 1:300 |
